# Effect of UV Irradiation (A and C) on *Casuarina equisetifolia*-Mediated Biosynthesis and Characterization of Antimicrobial and Anticancer Activity of Biocompatible Zinc Oxide Nanoparticles

**DOI:** 10.3390/pharmaceutics13111977

**Published:** 2021-11-22

**Authors:** Amna Komal Khan, Sullivan Renouard, Samantha Drouet, Jean-Philippe Blondeau, Iram Anjum, Christophe Hano, Bilal Haider Abbasi, Sumaira Anjum

**Affiliations:** 1 Department of Biotechnology, Kinnaird College for Women, Jail Road, Lahore 54000, Pakistan; aaykay28@gmail.com (A.K.K.); iram.anjum@kinnaird.edu.pk (I.A.); 2 Institut de Chimie et de Biologie des Membranes et des Nano-Objets, CNRS UMR 5248, Bordeaux University, 33600 Pessac, France; sullivan.renouard@u-bordeaux.fr; 3 Laboratoire de Biologie des Ligneux et des Grandes Cultures, INRAE USC1328, Eure et Loir Campus, University of Orleans, 28000 Chartres, France; samantha.drouet@univ-orleans.fr (S.D.); hano@univ-orleans.fr (C.H.); 4 Conditions Extrêmes et Matériaux—Haute Température et Irradiation (CEMHTI) CNRS UPR3079, 1D Avenue de la Recherche Scientifique, 45071 Orléans, France; jean-philippe.blondeau@univ-orleans.fr; 5 Department of Biotechnology, Quaid-i-Azam University, Islamabad 15320, Pakistan; bhabbasi@qau.edu.pk

**Keywords:** zinc oxide nanoparticles, UV-A, UV-C, caspase-3/7, mitochondrial membrane potential, brine shrimp lethality assay, ROS

## Abstract

The green synthesis of nanoparticles has emerged as a simple, safe, sustainable, reliable and eco-friendly protocol. Among different types of NPs, green-synthesized zinc oxide NPs (ZnONPs) show various promising biological uses due to their interesting magnetic, electrical, optical and chemical characteristics. Keeping in view the dependence of the therapeutic efficacy of NPs on their physico-chemical characteristics, the green synthesis of ZnONPs using *Casuarina equisetifolia* leaf extract under UV-A and UV-C light was carried out in this study. UV-irradiation helped to control the size and morphology of ZnONPs by exciting the electrons in the photoactive compounds of plant extracts to enhance the bio-reduction of ZnO into ZnONPs. *C. equisetifolia* leaf extract was found enriched with phenolic (2.47 ± 0.12 mg GAE/g DW) and flavonoid content (0.88 ± 0.28 mg QE/g DW) contributing to its 74.33% free-radical scavenging activity. FTIR spectra showed the involvement of polyphenols in the bio-reduction, stabilization and capping of ZnONPs. Moreover, SEM-EDX and XRD analyses showed great potential of UV-C light in yielding smaller (34–39 nm) oval-shaped ZnONPs, whereas UV-A irradiation resulted in the formation of fairly spherical 67–71 nm ZnONPs and control ZnONPs were of mixed shape and even larger size (84–89 nm). Green-synthesized ZnONPs, notably CE-UV-C-ZnONPs, showed promising anti-bacterial activities against *Bacillus subtilis, Pseudomonas fluorescens* and *Pseudomonas aeruginosa*. Moreover, ZnONPs also enhanced ROS production which led to a significant loss of mitochondrial membrane potential and activated caspase-3 gene expression and caspase-3/7 activity in human hepatocellular carcinoma (HepG2) cells. CE-UV-C-ZnONP treatment reduced HepG2 cell viability to as low as 36.97% owing to their unique shape and smaller size. Lastly, ZnONPs were found to be highly biocompatible towards brine shrimp and human red blood cells suggesting their bio-safe nature. This research study sheds light on the plausible role of UV radiation in the green synthesis of ZnONPs with reasonable control over their size and morphology, thus improving their biological efficacy.

## 1. Introduction

The green synthesis of nanoparticles has emerged as a simple, safe, sustainable, reliable and eco-friendly protocol [1]. Research studies focused on using plant extracts for the synthesis of NPs have intensified because plants are non-hazardous, easily available, provide a better yield and possess diverse phytochemicals. Numerous studies have been published on the use of diverse plant extracts for synthesizing different NPs via a green route [2]. Plant extracts are naturally enriched with phytochemicals which are able to reduce metal oxides and form NPs in a short time. Among these phytochemicals, flavonoids, alkaloids, terpenes, vitamins, polysaccharides and amino acids are important in reducing and stabilizing metal oxide NPs [3]. The exact mechanism of NP synthesis from plant extracts is not fully known but it generally consists of three main phases. Firstly, zinc acetate salt is converted into zinc hydroxide by reaction with NaOH [4]. Subsequently, during the activation phase, zinc hydroxide is bio-reduced and forms ZnO_2_^−2^ nuclei. The nucleated ZnO grows further to form ZnONPs with defined morphology during the growth phase which are subsequently stabilized by the phytochemicals that coat the NP surface during the final phase [5].

There are different types of metallic and metal oxide NPs synthesized via the green route, including silver, gold, copper, copper oxide, platinum, titanium dioxide, iron and zinc oxide [6]. Among these NPs, the plant-extract-assisted green synthesis of ZnONPs has been investigated widely [7,8]. However, the use of *Casuarina equisetifolia* extract for the green synthesis of ZnONPs has not been reported previously. *C. equisetifolia* is an evergreen tree commonly known as suru in Pakistan which exhibits several medicinal properties, as its bark helps in relieving headache, diarrhea, dysentery, toothache, fever, diabetes and cough [9]. Recently, the methanolic extracts of *C. equisetifolia* exhibited significant anti-oxidant and anti-arthritic properties owing to their rich polyphenol content [10]. Qualitative analysis has shown the presence of phytochemicals such as carbohydrates, glycosides, alkaloids, proteins, phenols, terpenoids, tannins and flavonoids in *C. equisetifolia* extract [11]. These phytochemicals have a crucial role during the green synthesis of NPs.

The morphology of NPs can be tuned by exposing the reaction mixture to UV radiation during the green synthesis of NPs. For example, gold nanoparticles (AuNPs) were synthesized from *Polyscias scutellaria* extract under UV light. The high energy UV radiation played a pivotal role in exciting the electrons in the active compounds of plant extracts to enhance the reduction of Au^+3^ ions. Stable AuNPs were synthesized with a size range of 5–20 nm [12]. The influence of UV radiation on the shape and size of AgNPs synthesized from *Aureobasidium pullulans* has also been evaluated in another study. As the irradiation time increased, the size of AgNPs decreased due to the breakdown of heated AgNPs into smaller NPs. Hence, UV radiation played a role in controlling the synthesis, distribution and size of nanoparticles [13]. However, such a radiation-controlled synthesis of ZnONPs has not been reported as yet. In this study, we report, for the first time, the effect of UV-A and UV-C illumination on the green synthesis of ZnONPs.

The biomedical uses of ZnONPs include drug delivery and antimicrobial, anti-cancerous and anti-diabetic activities [14,15,16,17]. Alternative treatment options are required due to rising multidrug resistant bacterial pathogens, and ZnONPs have received particular attention in this regard due to their extraordinary therapeutic properties [18]. Their high antibacterial activity is accredited to the large surface-area-to-volume ratio of NPs which enables the binding of a large number of ligands present on the NPs’ surface with bacterial receptors [19]. ZnONPs provide a safe and efficient treatment for drug resistant bacterial strains which are a major health challenge faced globally. Many research studies have shown the antibacterial activity of ZnONPs against a range of bacterial strains including *Bacillus subtilis*, *E. coli* and *Streptococcus aureus* [20]. Since ZnONPs can act as an alternate antibacterial agent, in our study we have evaluated the antibacterial potential of green-synthesized ZnONPs against Gram positive as well as Gram negative bacteria.

ZnONPs also exhibits anti-cancerous activities by preferentially killing cancer cells through an apoptotic mechanism via reactive oxygen species (ROS) generation [21]. Since conventional cancer treatments such as radiation and chemotherapy unfortunately show high level of toxicity to normal quickly dividing cells [22], there is a need to use alternate anti-cancer agents which are toxic towards tumor cells but safe to normal cells. Recently, Saeed et al. [23] reported the high cytotoxicity of green-synthesized ZnONPs against human liver carcinoma (HepG2) cells. In our study, we have analyzed and compared the cytotoxic potential of UV-mediated green-synthesized ZnONPs by measuring cell viability, mitochondrial membrane potential, intracellular ROS/RNS production and caspase-3/7 gene expression and activity in HepG2 cells.

All these applications require the administration of NPs in humans to exhibit their therapeutic effects and for this purpose safety is most critical. Therefore, green-synthesized NPs that are more safe and biocompatible should be a preferable option for biomedical applications [24]. This argument is supported by a report in which AgNPs were synthesized biologically using *Azadirachta indica* extracts as well as chemically. Compared to the chemically synthesized AgNPs, the green-synthesized AgNPs (94 nm) showed no appreciable change in cell viability, apoptotic change and ROS generation in normal HDFa cells. In addition to this, green-synthesized NPs were observed to be superior in anticancer activity against NCI-H460 cancerous cell line. Henceforth, biogenic NPs are normally non-toxic to normal cells [25]. In this article, we report the green synthesis of ZnONPs using *C. equisetifolia* leaf extract under UV-A and UV-C irradiation and evaluate their anti-bacterial and anti-cancerous biological activities and their bio-compatibility.

## 2. Materials and Methods

### 2.1. Preparation of Casuarina equisetifolia Leaf Extract

*Casuarina equisetifolia* leaves were freshly collected and identified by the Department of Botany, Kinnaird College for Women, Lahore. Firstly, 10 g of suru leaves were washed with running tap water three times and then with distilled water. After thorough washing, leaves were boiled in 350 mL distilled water until their volume was reduced to 100 mL. The mixture was finely crushed in pestle and mortar and filtered using Whatmann’s filter paper. Plant leaf extract was stored at 4 °C for further phytochemical analysis and ZnONP synthesis.

### 2.2. Phytochemical Analysis of Casuarina equisetifolia

#### 2.2.1. Total Phenolic Contents

Folin–Ciocalteu’s method was used to evaluate the total phenolic content (TPC) of *C. equisetifolia* leaf extract as described by Kamtekar et al. [26]. Firstly, 1 mL plant extract or gallic acid standard (1000, 750, 500, 250, 125, 50 μg·mL^−1^) was taken in a test-tube, to which 5 mL distilled water was added, followed by the addition of 0.5 mL Folin–Ciocalteu’s reagent. The mixture was shaken well. After 5 min incubation at RT, 1.5 mL of 20% sodium carbonate was added and the total volume was increased to 5 mL using distilled water. A deep blue color was observed. After 2 h of incubation at room temperature (RT), the calibration curve was plotted by measuring the absorbance of known gallic acid concentrations at 750 nm using a spectrophotometer (Analytik Jena, Specord 200 plus, Jena, Germany). The results for TPC were measured from a gallic acid calibration curve (y = 0.00004x + 0.0089, R^2^ = 0.9953) and expressed as mg GAE/g DW. TFC was calculated using the formula:

(1) $$\mathrm{TFC}\;\left(\mathrm{GAE}\right)\;=\frac{C\times V\;\left(\mathrm{mL}\right)}{m\;\left(g\right)}$$

where C = concentration of extract, V = volume of extract and m = mass of extract. The TPC was expressed as mg/g of gallic acid equivalents in milligrams per gram (mg·GAE/g) of dry extract. TPC was performed in triplicates.

#### 2.2.2. Total Flavonoid Contents

The aluminum chloride colorimetric method was adopted to measure total flavonoid content (TFC) as reported by Aryal et al. [27]. Briefly, 1 mL leaf extract or quercetin standard (25–200 μg·mL^−1^) was taken in a test-tube to which 0.2 mL of 1 M potassium acetate, 0.2 mL of 10% (*w*/*v*) AlCl_3_ solution and 5.6 mL distilled water were added, mixed well and incubated at RT for 30 min. The calibration curve was plotted by measuring absorbance of known quercetin concentrations at 415 nm using a spectrophotometer (Analytik Jena, Specord 200 plus). The results for TFC were measured from a quercetin calibration curve (y = 0.0057x + 0.0127, R^2^ = 0.9973) of quercetin (25–200 µg/mL) and expressed as quercetin equivalent (QE) per gram DW. TFC was calculated by using formula:

(2) $$\mathrm{TPC}\;\left(\mathrm{QE}\right)\;=\frac{C\times V\;\left(\mathrm{mL}\right)}{m\;\left(g\right)}$$

where C = concentration of extract, V = volume of extract and m = mass of extract. All the experimental work was run in triplicate.

#### 2.2.3. Free Radical Scavenging Activity (FRSA)

The antioxidant activity of *C. equisetifolia* leaf extract was evaluated by following the DPPH (2,2-Diphenyl-1-picrylhydrazyl) FRSA protocol as reported by Anjum et al. [28]. In short, 0.5 mL leaf extract and 4.5 mL DPPH (3.2 mg/100 mL methanol) were mixed together and incubated at RT for 1 h. Lastly, absorbance at 517 nm was measured using a spectrophotometer. All the experiments were run in triplicate. The FRSA was measured as a percentage of discoloration of DPPH using the equation stated below;

(3) $$\mathrm{FRSA}\;\left(\%\right)\;=100\times \left(1-\frac{A_{c}}{A_{s}}\right)$$

where A_c_ = absorbance of plant extract and DPPH and A_s_ = absorbance of DPPH solution (standard).

### 2.3. UV-Mediated Green Synthesis of ZnONPs

Control ZnONPs were synthesized via a green route according to the protocol of Anjum et al. [29] with minor changes. Briefly, 50 mL 0.02 M zinc acetate solution and 1 mL aqueous extract of *C. equisetifolia* leaves were mixed, while 2 M NaOH was added to it dropwise until the pH was 12. The mixture was constantly stirred using a magnetic stirrer for 2 h at RT under the illumination of UV-A (315–400 nm) and UV-C (200–280 nm) lamps and without any lamp as a control. Three types of NPs were synthesized, i.e., CE-ZnONPs (control), CE-UV-A-ZnONPs (UV-A-mediated green-synthesized ZnONPs) and CE-UV-C-ZnONPs (UV-C-mediated green-synthesized ZnONPs). A color change from colorless to yellowish and then white indicated the initiation of ZnONP synthesis. After 2 h, the ZnONP solution was micro-centrifuged at 6000 rpm for 15 min, the supernatant was discarded and the pellets were washed by re-suspending in distilled water. This step was performed three times and the pellets of ZnONP were dried at 40 °C overnight in an oven. Finally, the dried ZnONPs were crushed to a fine powder in a pestle and mortar.

### 2.4. Characterization of UV-Mediated ZnONPs

#### 2.4.1. UV-Visible Spectroscopy

The progress of the green synthesis of CE-ZnONPs, CE-UV-A-ZnONPs and CE-UV-C-ZnONPs was monitored using UV-visible spectroscopy after a 30 min interval during a 2 h reaction. The UV-Vis spectra of the ZnONP reaction mixture were recorded using a spectrophotometer (Analytik Jena, Specord 200 plus, Germany) in a wavelength range of 300–800 nm.

#### 2.4.2. Attenuated Total Reflection-Fourier Transform Infrared Spectroscopy (ATR-FTIR)

The Fourier-transform infrared (FTIR) spectra of the CE-ZnONPs, CE-UV-A-ZnONPs and CE-UV-C-ZnONPs were recorded by following the protocol described by Tungmunnithum et al. [30]. Reflectance spectra were acquired by a Burcker (Palaiseau, France) V70 interferometer in a reflectivity mode, which had an ATR accessory comprising of gold crystal. The wave numbers were measured within a 400 to 4500 cm^−1^ wavenumber range. The resolution of the instrument was about 4 cm^−1^. The FTIR data reported in this study are means of triplicates.

#### 2.4.3. Scanning Electron Microscopy (SEM) and Energy-Dispersive X-Ray (EDX) Analyses

The morphology of the CE-ZnONPs, CE-UV-A-ZnONPs and CE-UV-C-ZnONPs was evaluated by SEM—the SIGMA model (MIRA3; TESCAN, Brno, Czech Republic)—operating at an accelerating voltage of 10 kV. A dilute suspension of samples was dropped on a carbon tape and dried for 5 min under a mercury lamp and SEM images of ZnONPs at different magnifications were collected. EDX analysis was performed using an EDX detector attached to SEM for the elemental analysis of ZnONPs.

#### 2.4.4. X-Ray Diffraction Analysis

The crystalline nature of CE-ZnONPs, CE-UV-A-ZnONPs and CE-UV-C-ZnONPs was investigated by X-Ray diffraction (XRD). Briefly, ZnONPs were coated on an XRD grid and the measurements were performed in the scanning mode using an X-ray diffractometer (Shimadzu-Model, XRD6000), which was operated at 40 kV with a current of 30 mA and Cu/kα radiation in the range of 20–80° in 2θ angles. Moreover, the size of CE-ZnONPs, CE-UV-A-ZnONPs and CE-UV-C-ZnONPs was calculated by the Debye–Scherrer equation [31]:

(4) $$D=\frac{k\lambda }{\beta \;\cos\;\theta }$$

where k = shape factor (0.94); β = full width at half maximum (FWHM) in radians; λ = X-ray wavelength (λ = 1.5418 Å) and θ = Bragg’s angle.

### 2.5. Antibacterial Activity of Green-Synthesized ZnONPs

The antibacterial activities of CE-ZnONPs, CE-UV-A-ZnONPs and CE-UV-C-ZnONPs were evaluated against *Bacillus subtilis*, *Pseudomonas fluorescens* and *Pseudomonas aeruginosa* strains by using well-diffusion method [29]. Firstly, stock cultures of *B. subtilis*, *P. fluorescens* and *P. aeruginosa* were revived by streaking on nutrient agar. A total of 4.2 g of nutrient agar (NA) powder was dissolved in 150 mL distilled water to prepare NA and autoclaved at standard conditions. After autoclaving, NA was poured into sterilized petri plates and solidified. *B. subtilis*, *P. fluorescens* and *P. aeruginosa* were streaked to obtain a pure culture and incubated overnight at 37 °C in an incubator. Mueller Hinton agar (MHA) was prepared by dissolving 4.5 g MHA in 150 mL distilled water and autoclaved. Single colonies of *B. subtilis*, *P. fluorescens* and *P. aeruginosa* were picked from NA and swabbed thoroughly on MHA to assess antibacterial activity. Subsequently, 15 μL solutions of leaf extract (negative control), 0.02 M zinc acetate (positive control) and 10 mg/mL ZnONPs were added to the wells and ampicillin antibiotic discs (10 µg/disc) (Thermo Fisher Scientific™ Oxoid™) as standard were placed on the MHA plate. The plates were incubated overnight at 37 °C in an incubator followed by the measurement of the zone of inhibition at a millimeter scale.

### 2.6. Anti-Cancerous Activity of Green-Synthesized ZnONPs

#### 2.6.1. Cell Viability Assay by MTT

Human hepato-cellular carcinoma cells (HepG2) (ATCC HB-8065; American Type Culture Collection, Manassas, VA, USA) were cultured in Dulbecco’s Modified Eagle Medium for anti-cancerous studies. MTT (3-(4,5-dimethylthiazolyl-2)-2,5-diphenyltetrazolium bromide) dye was utilized to assess the cytotoxicity of CE-ZnONPs, CE-UV-A-ZnONPs and CE-UV-C-ZnONPs in vitro. The reduction of tetrazolium dye (MTT) by living cells into an insoluble purple-colored product (formazan) was recorded using a spectrophotometer as a measure of cell viability.

A quantity of 200 µg/mL of green-synthesized ZnONPs was added in a 96-well plate, which was pre-seeded with HepG2 cells (>90% viability; 1 × 10^4^ cells/well; 200 µL per well), for 24 h. A quantity of 10 µL MTT dye (5 mg/mL) was added per well and incubated for 3 h. Subsequently, 10% acidified sodium dodecyl sulfate was added to dissolve insoluble formazan. Lastly, after an overnight incubation of the cells, the plates were analyzed at 570 nm using a microplate reader (Platos R, 496. AMP, AMEDA Labordiagnostik GmbH, Graz, Austria). Non-treated cells (NTC) acted as a control. All the experiments were performed in triplicate. Cell viability was measured in terms of percentages with respect to control, using the equation below:

(5) $$\mathrm{Viability}\;\left(\%\right)\;=\frac{\mathrm{Ab}\;\mathrm{of}\;\mathrm{sample}-\;\mathrm{Ab}\;\mathrm{of}\;\mathrm{control}}{\mathrm{Ab}\;\mathrm{of}\;\mathrm{NTC}-\;\mathrm{Ab}\;\mathrm{of}\;\mathrm{media}}\times 100$$

#### 2.6.2. Measurement of Intracellular Reactive Oxygen and Nitrogen Species (ROS/RNS)

The intracellular ROS/RNS level was assessed by using dihydrorhodamine-123 (DHR-123) fluorescent dye (Sigma-Aldrich, Saint Quentin Fallavier, France) as reported by Nazir et al. [32]. HepG2 cells in a pre-seeded 96-well plate (>90% viability; 1 × 10^4^ cells/well; 200 µL per well) were washed twice with phosphate-buffered saline (PBS), then resuspended in PBS containing 0.4 μM DHR-123 and incubated for 10 min at 30 °C in dark. Lastly, the fluorescence signal (λex = 505 nm, λem = 535 nm) was recorded on VersaFluor Fluorimeter (Biorad, Marnes la Coquette, France).

#### 2.6.3. Measurement of Mitochondrial Membrane Potential (MMP)

The MMP (ΔΨm) of HepG2 cells treated with green-synthesized ZnONPs was measured with 3,3′-dihexyloxacarbocyanine iodide (DiOC_6(3)_, Sigma) which works by staining mitochondria according to their MMP. Cells were incubated in culture media supplied with 25 nM DiOC_6(3)_ at 37 °C for 40 min. MMP was expressed as relative fluorescent units (RFU). All the experiments were performed three times.

#### 2.6.4. Caspase-3 Gene Expression and Caspase-3/7 Activity

For the measurement of caspase-3 gene expression, total RNA was isolated and quantified using the GeneJET RNA Purification Kit (Thermo, Illkirch-Graffenstaden, France) and Quant-iT RNA Assay Kit (Invitrogen), respectively. A first-strand cDNA synthesis kit (Thermo) was used to perform reverse transcription. A PikoReal real time PCR system (Thermo, Illkirch-Graffenstaden, France) was used to perform quantitative PCR using the DyNAmoColorFlash SYBR Green qPCR Kit (Thermo, Illkirch-Graffenstaden, France). A 10 µL quantity of PCR reaction mixture was made that contained 0.5 µL of diluted cDNAs, 2 × SYBR Green Mix and 1 µM of each of the primer pairs. PCR reaction was carried out as follows: 7 min at 95 °C, 40 cycles of 10 s at 95 °C, 10 s at 60 °C and 30 s at 72 °C. Data were analyzed with Pikoreal software (Thermo, Illkirch-Graffenstaden, France). Three biological replicates and two technical repetitions were performed for each sample. The caspase-3 primers used were: 5′-TGTTTGTGTGCTTCTGAGCC-3′ (forward primer) and 5′-CACGCCATGTCATCATCAAC-3′ (reverse primer) (amplicon size: 210 bp).

Caspase-3/7 activity was measured by using frozen cells to obtain cytosolic protein extracts. A glass chilled pestle and mortar was used to ground the samples in 500 mL ice-cold extraction buffer that contained 10% (*w*/*v*) sucrose, 100 mM HEPES (pH 7.2), 5 mM DTT, 1% (*v*/*v*) NP40 and 0.1% (*w/v*) CHAPS. The incubation of the homogenate was carried out on ice for 15 min and centrifuged twice at 13,000× *g* for 10 min at 4 °C to pellet out cell debris, and the supernatant was filtered through a 0.22 mm filter. An Apo-ONE Homogeneous Caspase-3/7 Assay kit (Promega, Charbonnières-les-Bains, France) was used to measure in vitro caspase-3/7 activity following the instructions of manufacturer.

### 2.7. Biocompatibility Studies

#### 2.7.1. Brine Shrimp Lethality Assay

The lethality of CE-ZnONPs, CE-UV-A-ZnONPs and CE-UV-C-ZnONPs (20 mg/mL stock in water) against *Artemia salina* (brine shrimp) was evaluated in a 96-well plate (300 µL) for 24 h. The larvae of the brine shrimp were used for toxicological study by following the protocol reported by Ahmed et al. [33]. Briefly, the eggs of *A. salina* were placed in a specifically designed plastic tray with two compartments containing sterile sea water (38 g/L) supplemented with 6 mg/L dried yeast. The eggs were hatched by incubating for a period of 24–48 h, with a constant supply of oxygen. Proper illumination was ensured to maintain the temperature (30–32 °C) and light necessary for hatching. A total of 10 mature nauplii (phototropic) were picked with a Pasteur pipette and placed into the wells, to which ZnONPs (25, 50, 100, and 200 µg/mL) were added, and the final volume was adjusted to 300 µL. A quantity of 1% DMSO in sea water acted as a negative control while doxorubicin (1–10 µg/mL) served as a positive control. After 24 h incubation, live shrimp were quantified and the median lethal concentration (LC50) was measured using table curve 2D v5.01 of the test extracts with ≥50% mortality.

#### 2.7.2. Biocompatibility with Human Red Blood Cells (hRBCs)

The biocompatibility of CE-ZnONPs, CE-UV-A-ZnONPs and CE-UV-C-ZnONPs was evaluated against freshly isolated hRBCs. Blood samples of 1 female and 2 male (average age 28 years) were withdrawn using sterile syringes after obtaining consent. The procedures dealing with human subjects were carried out in accordance with the ethical standards of the International and National Research Committees and with the 1964 Helsinki Declaration and its later amendments. Blood was collected in tubes containing EDTA to prevent blood clotting [34]. RBCs were extracted by centrifuging 1 mL blood for 5 min at 14,000 rpm. A quantity of 200 µL of pelleted erythrocyte was shaken in 9.8 mL of PBS (pH: 7.2). In a 1.5 mL Eppendorf tube, 100 µL of ZnONPs and erythrocytes was taken, incubated at 35 °C for 1 h and centrifuged for 10 min at 10,000 rpm. In a 96-well plate, 100 µL of supernatant was added and the hemoglobin released was measured at 540 nm by a BioTek ELX800 Absorbance Microplate Reader (BioTek Instruments, Comar, France). Triton X-100 and DMSO served as a positive and negative control, respectively. The results were measured as % hemolysis using the following formula:

(6) $$\%\;\mathrm{Hemolysis}\;=\;\frac{\mathrm{Abs}\;\mathrm{of}\;\mathrm{Sample}\;-\;\mathrm{Abs}\;\mathrm{of}\;\mathrm{Negative}\;\mathrm{control}}{\mathrm{Abs}\;\mathrm{of}\;\mathrm{Positive}\;\mathrm{Control}-\;\mathrm{Abs}\;\mathrm{of}\;\mathrm{Negative}\;\mathrm{Control}}\;\times 100$$

### 2.8. Statistical Data Analysis

All the data was evaluated statistically to find out average values and standard deviation by SPSS (Windows Version 7.5.1, SPSS Inc., Chicago, IL, USA). The data were expressed as mean ± SD.

## 3. Results

### 3.1. Phytochemical Analysis of Casuarina equisetifolia

#### 3.1.1. Total Phenolic Contents (TPC) of *C. equisetifolia*

Phenolic compounds possess redox properties responsible for antioxidant activity, thus acting as reducing agents of metal ions during NP synthesis [35,36]. The TPC of *C. equisetifolia* leaf extract was measured to be 2.466 ± 0.12 mg·GAE/g DW. This value is approximately close to 3.67 ± 0.30 mg·GAE/g as reported by Brist et al. for *C. littorea* [37]. Saranya et al. [11] reported 43.2 mg·GAE/g TPC for the bark extract of *C. equisetifolia*, suggesting a high quantity of phenolic compounds in bark. Our findings show an enriched content of phenolics present in *C. equisetifolia* which can be helpful in effectively reducing and stabilizing ZnONPs during synthesis.

#### 3.1.2. Total Flavonoid Contents (TFC) of *C. equisetifolia*

Flavonoids belong to a group of phenolic compounds with industrial significance [38]. They act as chelating agents of metal oxide as they possess a functional hydroxyl group which mediates antioxidant activity [39]. The TFC of *C. equisetifolia* leaf extract was found to be 0.878 (SD ± 0.28) mg·QE/g. These findings are in accordance with 0.118 ± 0.001 mg of QE/g as reported by Brist et al. [37]. However, Saranya et al. reported the TFC of *C. equisetifolia* bark extract as 27.86 ± 0.23 mg of QE/g [11].

### 3.2. Free Radical Scavenging Activity (FRSA)

The TPC and TFC assay determines only the phenolic and flavonoid content which is not representative of total antioxidant activity of all the constituents of the extract under study. Therefore, FRSA helps to determine the antioxidant potential of all the constituents present in plant extract [40]. FRSA is a rapid, easy and sensitive method to evaluate the antioxidant potential of plant extract where DPPH scavenges the free radicals [41]. We found the FRSA of the leaf extract of *C. equisetifolia* to be 74.33 ± 0.21%. This value is closer to 80% FRSA of the bark extract of *C. equisetifolia* at an 80 µg/mL concentration of DPPH as reported by Saranya et al. [11]. Overall, the results suggest the presence of enriched flavonoid and phenolic compounds in suru which contribute to better antioxidant activity and consequently good yields of nanoparticles.

### 3.3. Characterization of ZnONPs

#### 3.3.1. UV-Visible Spectroscopy

**Figure 1:**
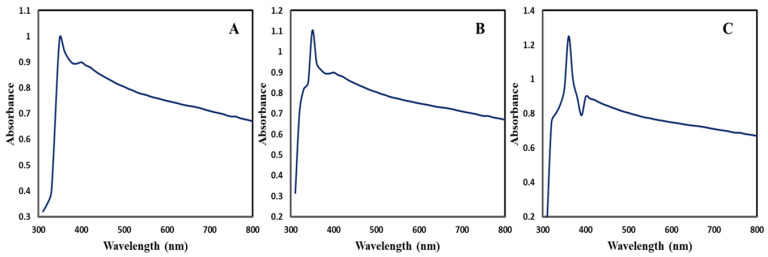
UV-Vis spectra of ZnONPs (**A**) CE-ZnONPs (**B**) CE-UV-A-ZnONPs (**C**) CE-UV-C-ZnONPs.

The green synthesis of ZnONPs was monitored by measuring UV-visible spectra in the range of 300–800 nm wavelength. CE-ZnONP, CE-UV-A-ZnONP and CE-UV-C-ZnONP reaction mixtures showed strong peaks between 350–360 nm which are attributed to their large excitation binding energy at room temperature as depicted in Figure 1A–C.

Our results show similarity with the reports of Fakhri et al. [31] in which ZnONPs synthesized from *Laurus nobilis* showed a distinct peak centered around 350 nm. Similar to our results, green-synthesized ZnONPs from *Solanum torvum* showed an absorption peak at 359 nm, which is a characteristic peak of ZnONPs [42]. In our study, CE-UV-C-ZnONPs displayed a sharp and narrow peak at 360 nm while CE-UV-A-ZnONPs absorbed maximum UV-radiation at 350 nm. Lastly, CE-ZnONPs showed weak surface plasmon resonance (SPR) at 350 nm. For these findings, we can conclude that due to the small size of CE-UV-C-ZnONPs, they showed better absorbance, hence displaying a narrow and sharp peak, while CE-ZnONPs displayed poor absorption which could be due to their larger size and decreased particle density [43].

#### 3.3.2. Attenuated Total Reflection-Fourier Transform Infrared Spectroscopy (ATR-FTIR)

**Figure 2:**
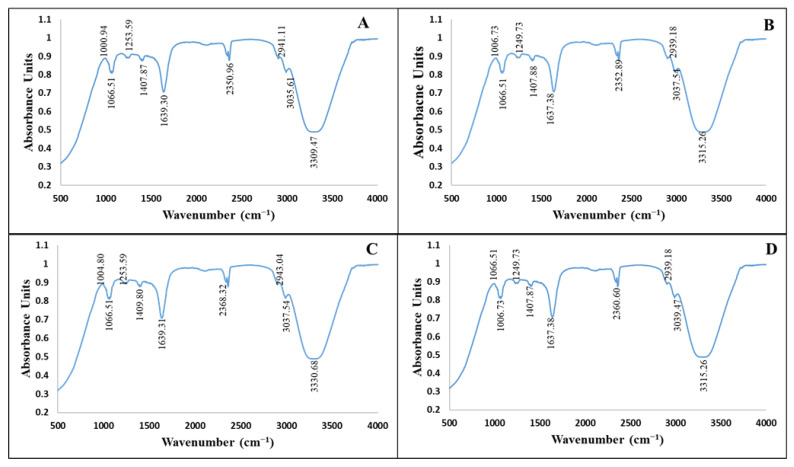
FTIR spectra of ZnONPs. (**A**) *Casuarina equisetifolia* leaf extract (**B**) CE-ZnONPs (**C**) CE-UV-A-ZnONPs (**D**) CE-UV-C-ZnONPs.

FTIR analysis helped in identifying various phytochemicals from *C. equisetifolia* leaf extract which possibly helped in the reduction and stabilization of NPs. The FTIR spectrum for *C. equisetifolia* leaf extract showed intense peaks at ~3309.47 cm^−1^, 3035.61 cm^−1^, 2941.11 cm^−1^, 1639.31 cm^−1^, 1407.87 cm^−1^, 1253.59 cm^−1^, 1066.51 cm^−1^ and 1000.94 cm^−1^ (Figure 2A). These peaks correspond to stretching vibrations of –O–H (phenol), –C=O (carboxylic acids), –C–C (aromatics), –C–N (aliphatic amines) and =C–H bend (alkenes). Similar peak intensities were observed for CE-ZnONPs, CE-UV-A-ZnONPs and CE-UV-C-ZnONPs (Figure 2B–D) which suggests the involvement of polyphenols, alkaloids and alcohols as reducing and capping agents. FTIR characterization displayed a large number of peaks, which suggests the presence of complex phytochemicals such as alcohols, phenols, terpenoids, alkenes and aromatic compounds in the plant extracts and their involvement in the bioreduction and capping of ZnONPs. The peak observed at 1638 cm^−1^ reflects C=O functional group stretching which is in accordance with the literature [44]. The absorption peaks in the range 3300–2500 cm^−1^ correspond to the O–H stretching of carboxylic acid, also described by Fahimmunisha et al. [45]. The peaks in the range 1066–1250 cm^−1^ represent C–N stretch aliphatic amines which are a characteristic of proteins/enzymes [46]. The amine group suggests the ability of amino acids or proteins to bind to a metal and form a layer around it to prevent agglomeration, hence stabilizing the NPs. Minor peaks at 3037.54 cm^−1^ and 3039.47 cm^−1^ show the vibrational bond of =C–H stretch assigned to alkenes [47]. The FTIR analysis reveals that green-synthesized ZnONPs were not only surrounded by polyphenols, flavonoids and terpenoids but also proteins. These phytochemicals were also responsible for the reduction of ZnO to ZnONPs by oxidizing aldehydic groups to carboxylic acids [48].

#### 3.3.3. Scanning Electron Microscopy (SEM) Analysis

**Figure 3:**
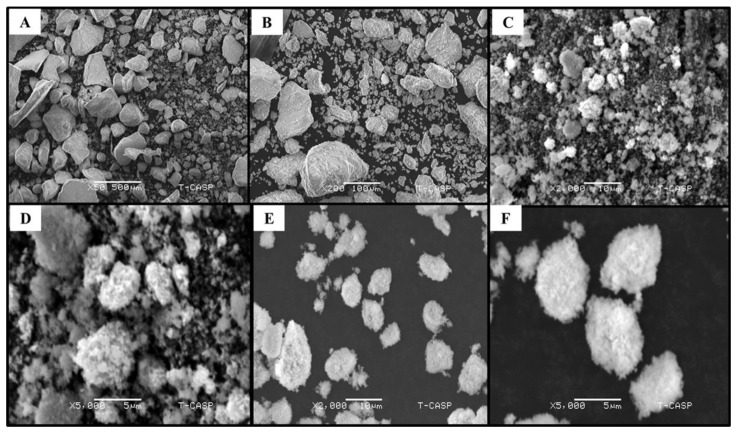
SEM images of ZnONPs. (**A**,**B**) CE-ZnONPs at ×50 magnification (500 µm) and ×200 (100 µm) (**C**,**D**) CE-UV-A-ZnONPs at ×2000 magnification (10 µm) and ×5000 (5 µm) (**E**,**F**) CE-UV-C-ZnONPs at ×2000 magnification (10 µm) and ×5000 (5 µm).

SEM was used to determine the shape and structure of green-synthesized ZnONPs. The SEM analysis of CE-ZnONPs without any UV light irradiation revealed irregular and mixed shapes (Figure 3A,B), whereas SEM images suggested that UV-A light influenced the green synthesis of ZnONPs by forming dominantly spherically-shaped ZnONPs (Figure 3C,D). Similarly Khan et al. [13] reported the formation of spherically shaped pullulan-mediated silver nanoparticles (AgNPs) when exposed to UV-A light (365 nm) for 96 h. The UV-C light promoted the formation of predominantly oval-shaped ZnONPs (Figure 3E,F). Moreover, spherically shaped silver and gold NPs were produced from Cornelian cherry fruit extract promoted by 365 nm UV-light for 2.5 h [49]. Similar results were obtained in a study where spherically shaped ZnONPs biosynthesized from *P. caerulea* extract were formed when zinc acetate was used as a precursor salt [50]. Pan et al. [51] also reported the formation of similarly shaped ZnONPs. In our study, we used 1 ml plant leaf extract for ZnONP synthesis. In future, the concentration of extract used could be varied to obtain more refined and distinct shapes.

The formation of agglomerated spherically shaped AgNPs by the UV irradiation method have been reported previously [52]. However, there is no report which involves the *C. equisetifolia* leaf-extract-mediated synthesis of ZnONPs under the influence of UV-A and UV-C radiation. These findings reveal that UV radiation play a key role in determining the morphology of NPs and defining their shape.

#### 3.3.4. Energy-Dispersive X-Ray (EDX) Analysis

**Figure 4:**
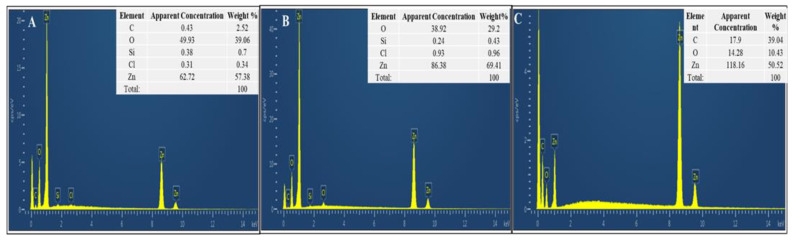
EDX spectra of ZnONPs (**A**) CE-ZnONPs (**B**) CE-UV-A-ZnONPs (**C**) CE-UV-C-ZnONPs.

The elemental composition of NPs was confirmed by EDX. Figure 4A–C shows an EDX spectrum which confirms the presence of zinc and oxygen in powder samples of CE-ZnONPs, CE-UV-A-ZnONPs and CE-UV-C-ZnONPs. Furthermore, ZnONPs were of high quality and free of any impurities as no characteristic peak of any other element was detected. Minor signals from C are likely due to X-ray emission from biomolecules capping the ZnONPs. The EDX weight % of Zn and O in CE-ZnONPs was 57.3% and 39.06%, respectively. Zn and O content in the CE-UV-A-ZnONP sample was found to be 69.41% and 29.2%, respectively, and in the CE-UV-C-ZnONP sample 50.52% and 10.43%, respectively, showing a marked difference in stoichiometric mass percent of Zn and O in different ZnONP samples. Other studies also showed the characterization of elemental composition of ZnONPs and their purity using EDX [53,54].

#### 3.3.5. X-Ray Diffraction Analysis

**Figure 5:**
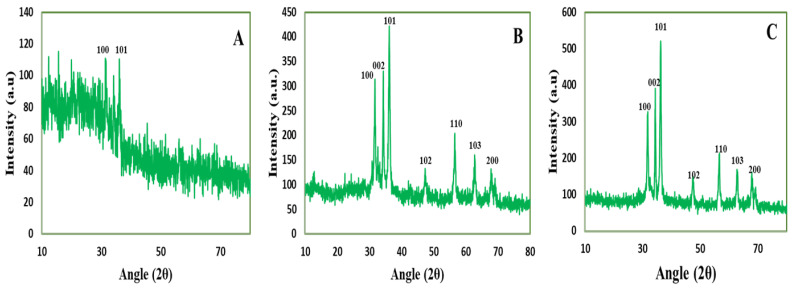
XRD patterns of ZnONPs (**A**) CE-ZnONPs (**B**) CE-UV-A-ZnONPs and (**C**) CE-UV-C-ZnONPs.

The crystalline nature of CE-ZnONPs, CE-UV-A-ZnONPs and CE-UV-C-ZnONPs was analyzed by XRD. The CE-ZnONPs showed scattering of X-rays in many directions that means the ZnONPs synthesized without UV irradiation were not of defined crystallinity (Figure 5A). However, the XRD diffractogram observed at 2θ for the CE-UV-A-ZnONPs showed characteristic peaks at 31.75°, 34.30°, 36.05°, 47.25°, 56.75°, 62.55° and 67.90° (Figure 5B), which correspond to the 100, 002, 101, 102, 110, 103 and 200 reflection planes and the hexagonal structure of ZnONPs (JCPDS 36-1451) [55]. Similarly, the XRD diffractogram of CE-UV-C-ZnONPs showed diffraction peaks at 31.65°, 34.55°, 36.10°, 47.70°, 56.45°, 63.00° and 68.1° (Figure 5C), which correspond with the 100, 002, 101, 102, 110, 103, and 200 reflection planes and the hexagonal structure of ZnONPs. These results confirm the crystallinity of ZnONPs synthesized under the influence of UV-irradiation. Likewise Abbasi et al. reported the hexagonal wurtzite structure of ZnONPs synthesized by in vitro callus and root extracts of *Linum usitatissimum* [29].

The average particle size of CE-ZnONPs, CE-UV-A-ZnONPs and CE-UV-C-ZnONPs was calculated to be 84–89 nm, 67–71 nm and 34–39 nm, respectively, using the Scherrer equation as also reported by Gawade et al. [56]. The ZnONPs synthesized under the illumination of UV-C were of smaller size which is due to the high energy of UV-C, which therefore efficiently reduces ZnO into ZnONPs. In one study, the effect of UV radiation on the size and shape of AgNPs synthesized from pullulan was evaluated. The AgNO_3_ solution and pullulan mixture was exposed to UV-C (200–280 nm) for times ranging from 1–96 h. As the irradiation time increased, a reduction in the size of AgNPs (42.27 nm after 48 h of UV irradiation) was observed due to the breakdown of heated AgNPs into smaller NPs [13]. As the physico-chemical characteristics of NPs greatly determine their applications; for example, ZnONPs smaller than 100 nm are thought to be more biocompatible than larger ones [57]. Our results showed that the UV light positively affected the tuning of the size and crystalline nature of CE-UV-A-ZnONPs and CE-UV-C-ZnONPs which can be used for effective therapeutic applications.

### 3.4. Antibacterial Activity of Green-Synthesized ZnONPs

NPs serve as alternative agents to counter the rising antibiotic resistance of bacteria [58]. NPs have the ability to strongly interact with the negatively charged bacterial membrane, leading to a change in membrane permeability and inducing oxidative stress which ultimately kills the bacterial cells [59]. Among various reported NPs, ZnONPs were found to be safe for human use, allowing their use as bactericidal agents which are biocompatible with human cells [60]. Therefore, in our study we assessed the antibacterial activity of CE-ZnONPs, CE-UV-A-ZnONPs and CE-UV-C-ZnONPs against *B. subtilis*, *P. fluorescens* and *P. aeruginosa*. The zone of inhibition (mm) was calculated to assess the extent of antibacterial activity. *C. equisetifolia* leaf extract (CE-LE) acted as a negative control so no significant zone of inhibition was formed in all MHA plates, whereas zinc acetate served as a positive control.

**Figure 6:**
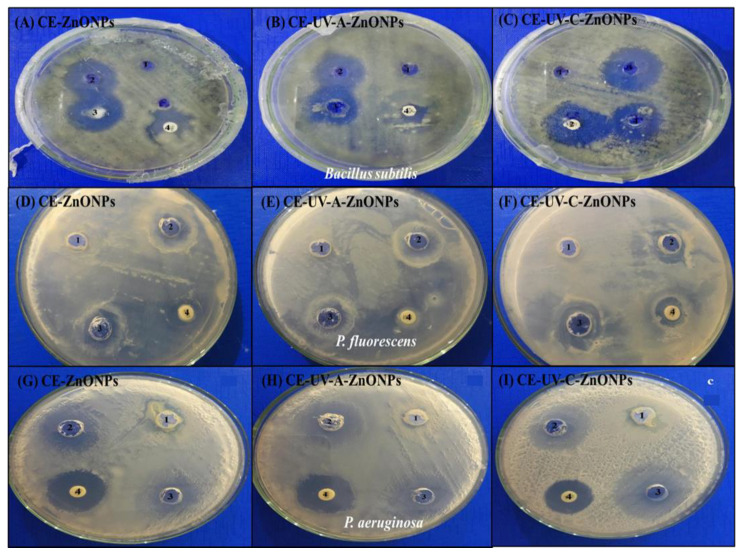
Antibacterial activity of ZnONPs against (**A**–**C**) *B. subtilis, (***D**–**F**) *P. fluorescens* (**G**–**I**) *P. aeruginosa*. In each petri plate, well 1 contains *Casuarina equisetifolia* leaf extract, well 2 contains zinc acetate, well 3 contains ZnONPs and well 4 contains an ampicillin disc.

A number of studies have reported the efficacy of ZnONPs as antibacterial agents against Gram negative as well as Gram positive bacteria [61,62,63]. Our study revealed that all types of synthesized ZnONPs exhibited significant antibacterial activity against *B. subtilis*, even greater than the standard antibiotic disc. Moreover, CE-UV-C-ZnONPs formed the greatest zone of inhibition (12 mm (Figure 6C)) followed by CE-ZnONPs (11 mm (Figure 6A)) and the least zone of inhibition was formed by CE-UV-A-ZnONPs (10 mm (Figure 6B)). Ampicillin (Amp) discs formed a zone of inhibition of less than 10 mm. Therefore, it can be concluded that ZnONPs formed under UV-irradiation have superior antibacterial activity than commercial antibiotics. Additionally, CE-UV-C-ZnONPs are more potent antibacterial agents due to their small size and the oval shape of NPs synthesized under UV-C illumination.

Figure 6D–F shows the antibacterial activity of ZnONPs against *Pseudomonas fluorescens*. *P. fluorescens* are Gram-negative, unicellular and non-spore-forming rods, which are known to contaminate ready-to-eat foods [64,65]. A notable spoilage case includes a blue mozzarella cheese event in Italy, where blue stains on mozzarella cheese were observed. Upon testing 70,000 cheese chunks, *P. fluorescens* was found to be responsible for the cheese spoilage [66]. The present study showed the promising antibacterial activity of green-synthesized ZnONPs against *P. fluorescens*; more significantly, CE-UV-C-ZnONPs formed a greater zone of inhibition of 9.5 mm (Figure 6F, Table 1), while CE-UV-A-ZnONPs formed an 8.5 mm (Figure 6E, Table 1) and CE-ZnONPs formed a 9 mm zone of inhibition (Figure 6D, Table 1). Our results are in agreement with previous reports showing the sensitivity of *P. fluorescens* to TiO2/ZnO nanoparticles supported in 4A zeolite [67].

*P. aeruginosa* is another Gram-negative, rod-shaped bacterium belonging to the *Pseudomonadaceae* family which is a ubiquitously distributed opportunistic pathogen. *P. aeruginosa* poses a hazard as a nosocomial infection, especially in immunocompromised and critically ill patients. Moreover, *P. aeruginosa* is increasingly becoming drug-resistant, hence causing higher mortality [68]. To counter this emerging drug resistance problem, ZnONPs have emerged as a novel and effective antibacterial agent. We found that CE-UV-C-ZnONPs formed the highest zone of inhibition (15 mm) against *P. aeruginosa* (Figure 6I) followed by CE-UV-A-ZnONPs, forming a 12 mm (Figure 6H, Table 1), and CE-ZnONPs, forming a 10.5 mm zone of inhibition (Figure 6G, Table 1). Similar to our findings, Tayel et al. [69] reported the formation of a 17 mm zone of inhibition against *P. aeruginosa* and an 18 mm against *P. fluorescens* at 26 mM and 24 mM minimum inhibitory concentrations of ZnONPs, respectively.

Our findings show the excellent antibacterial activity of ZnONPs, even better than that of an antibiotic disc. Notably, UV-C-mediated green-synthesized ZnONPs exhibited better bactericidal activity than control and UV-A-mediated ZnONPs. This could be due to the difference in size and the shape of ZnONPs. Hence, the ovoid-shaped CE-UV-C-ZnONPs have a potential to be used as antibacterial agents in future.

### 3.5. Anti-Cancerous Activities of Green-Synthesized ZnONPs

#### 3.5.1. Cell Viability Assay by MTT

**Figure 7:**
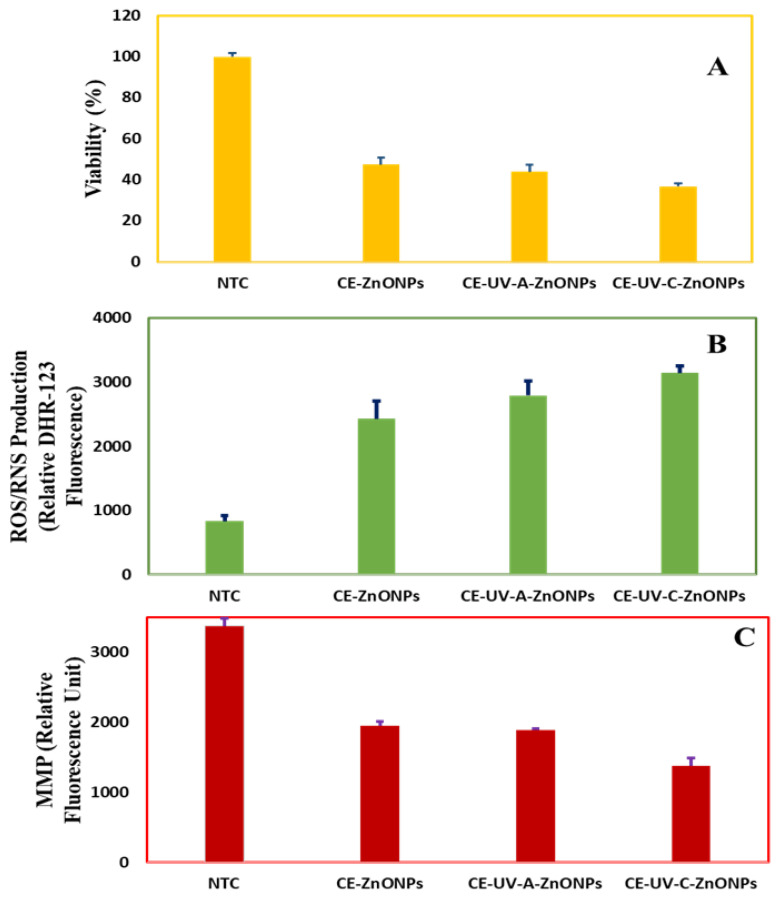
Anti-cancerous activities of ZnONPs (**A**) Measurement of HepG2 cell viability (**B**) Production of ROS/RNS (**C**) Measurement of mitochondrial membrane potential of HepG2 cells in response to green-synthesized-ZnONP treatment.

The cytotoxic ability of green-synthesized ZnONPs was evaluated against the HepG2 cell line by using a colorimetric MTT assay as reported by Abbasi et al. [70]. Cell viability was assessed as a measure of the cytotoxic/anti-proliferative ability of ZnONPs. Cell viability is the number of live cells expressed as a percentage of control [71]. Our analysis showed that all types of CE-mediated ZnONPs were cytotoxic towards HepG2 cells as compared to non-treated cells (NTC). The viability of NTC was found to be 100 ± 1.71%. On the other hand, cell viability was 47.57 ± 3.32% when treated with CE-ZnONPs and 44 ± 3.36% when treated with CE-UV-A-ZnONPs. Notably, CE-UV-C-ZnONP-treated cells were least viable (36.97 ± 1.53%—Figure 7A). Our results are in agreement with previous reports where green-synthesized ZnONPs were found to be cytotoxic towards HepG2 cells [70].

Chen et al. [72] studied the size dependent cytotoxicity of ZnONPs towards HepG2 cells. They showed that smaller-sized ZnONPs (20 nm) had a greater cytotoxic effect at all concentrations (0–160 mg/L) as compared to larger sizes, i.e., 90 nm and 200 nm ZnONPs. Our results also support this and show the promise of ZnONPs as a cytotoxic agent against liver cancer cells, especially that of CE-UV-C-ZnONPs, which could be attributable to their smaller size and unique shape.

#### 3.5.2. Measurement of Intracellular ROS/RNS

Dihydrorhodamine 123 (DHR123) is a non-toxic and non-fluorescent dye which penetrates the cell membrane where it is converted into a positively charged fluorescent rhodamine 123 by ROS and consequently stains mitochondria [73]. In our study, we found that the ROS level in non-treated cells was 835 ± 80.17 relative DHR-123 fluorescence whereas ZnONP-treated cells showed a significant increase in intracellular ROS level (Figure 7B). CE-ZnONPs produced 2430 ± 277.3 relative DHR-123 fluorescence, CE-UV-A-ZnONPs produced 2790 ± 221.50 relative DHR-123 fluorescence and CE-UV-C-ZnONPs led to the highest ROS level: 3142 ± 106.52 relative DHR-123 fluorescence. These results show an inverse relation between higher levels of ROS production, hence a lower cell viability. Wang et al. [74] reported similar results against LTEP-a-2 Cells, where 1.5 µg/mL ZnONPs led to a higher ROS level (1200 RFU) after 2 h. These findings show the unique potential of ZnONPs particularly synthesized under UV illumination as anti-cancerous agents by inducing intracellular ROS production at greater levels.

#### 3.5.3. Measurement of Mitochondrial Membrane Potential (MMP)

Mitochondria are a main site where intracellular ROS is produced, therefore the depletion of anti-oxidants as well as an increase in ROS level leads to a mitochondrial damage. An increase in ROS levels has been linked with a loss of mitochondrial membrane potential (MMP) [75]. We used 3,3′-dihexyloxacarbocyanine iodide cationic fluorochrome to measure the MMP of HepG2 cells in response to treatment with ZnONPs. A significant loss of MMP of HepG2 cells when treated with CE-UV-C-ZnONPs (1371.97 ± 118.87 RFU) was recorded, whereas treatment with CE-UV-A-ZnONPs showed a loss of MMP measured to be 1883.73 ± 27.40 RFU and the lowest value was reported for cells treated with CE-ZnONPs 1947.40 ± 60.91 RFU (Figure 7C). However, no significant loss of MMP was reported for control (NTC). Chen et al. [76] also supports our results where fungal-derived ZnONPs showed a loss of MMP in HeLa cells via ROS-triggered mitochondrial-pathway-mediated MMP reduction. Therefore, ZnONPs have been found to possess significant apoptosis inducing and anti-proliferative activity, which is enhanced due to the unique shape and smaller size of CE-UV-C-ZnONPs.

#### 3.5.4. Caspase-3 Gene Expression and Caspase-3/7 Activity

**Figure 8:**
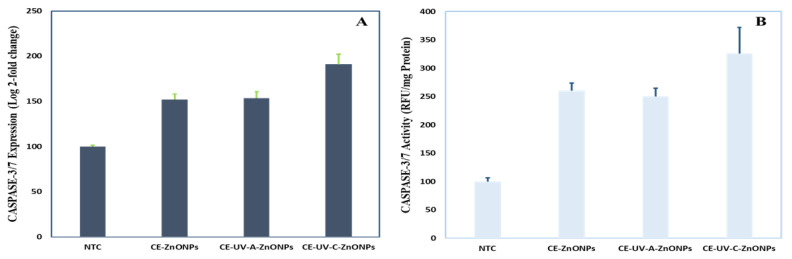
(**A**) Caspase3 gene expression (**B**) Caspase3/7 activity in response to ZnONP treatment of HepG2 cells.

Apoptosis is a programmed cell death brought about by the synchronized action of caspases [77]. The reduction of MMP disrupts mitochondrial outer membrane pores and releases apoptotic caspases [78]. The exposure of HepG2 cells to green-synthesized ZnONPs elevated the level of caspase-3 gene expression and caspase-3/7 activity. As compared to control (NTC cells, 100 ± 1.81) CE-ZnONPs (152 ± 6.26) and CE-UV-A-ZnONPs (153.80 ± 7.17) slightly increased caspase-3 gene expression. A more significant stimulation of caspase-3 gene expression was recorded for CE-UV-C-ZnONPs (190.97 ± 11.38) (Figure 8A). Similarly, CE-UV-C-ZnONPs stimulated the caspase-3/7 activity to the highest value, i.e., 325.53 ± 46.28 RFU/mg protein as compared to NTC 100 ± 6.61 RFU/mg protein. An intermediate increase in caspase-3/7 activity was recorded when treated with CE-ZnONPs 260.20 ± 13.67 RFU/mg protein, whereas treatment with CE-UV-A-ZnONPs resulted in 249.73 ± 14.70 RFU/mg protein elevation (Figure 8B). Previous findings also suggest the anti-cancerous potential of green-synthesized ZnONPs in enhancing the activity of apoptotic caspases. Recently, Duan et al. [79] reported the elevation of caspase-3, 8 and 9 in human melanoma cells (A375) when exposed to ZnONPs synthesized from *Cardiospermum halicacabum*.

The chemotherapeutic drugs available cause serious side effects to human health. Consequently, alternative, safe and effective anti-cancerous agents are required [80]. ZnONPs are one such nano-formulation known to exhibit cytotoxic effects against a number of cancer cells [76]. A possible mechanism for the anti-cancerous activity of ZnONPs is proposed. The first step during ZnONPs cytotoxicity against cancer cells is the internalization of NPs most probably via the endocytosis pathway. Smaller sized NPs (approx. 10 nm) are said to coat the plasma membrane prior to their incorporation whereas larger NPs (100 nm) are directly internalized without accumulating onto the cell membrane [81]. The major endocytosis mechanisms for NP uptake are phagocytosis, diffusion, pinocytosis and clathrin/caveolae-mediated endocytosis [82]. Factors such as NP size, morphology, surface chemistry and incubation environment affect the uptake and internalization of NPs [72].

Once internalized, ZnONPs mediate cytotoxicity through several proposed mechanisms. ROS and free radical generation is the key mechanism of NP toxicity which induces oxidative stress [83]. In our study, we found an enhanced level of ROS production in HepG2 cells when exposed to 200 µg/mL of green-synthesized ZnONPs. The accumulation of ROS alters the health of cells by inhibiting the Atpase-2 enzyme, which leads to the influx of extracellular Ca^+2^ ions [84]. Increase in intracellular Ca^+2^ ions leads particularly to mitochondrial dysfunction, DNA damage and fragmentation [85]. Mitochondria is the powerhouse as well as arsenal in cells, where cell death by apoptosis and non-apoptosis mechanisms is triggered, which results in the disruption of electron transport, adenosine triphosphate production, oxidative phosphorylation, release of caspase protease and alteration in cellular reduction–oxidation potential [86]. The increase in the intracellular ROS level opens a mitochondrial transition pore which decreases MMP, initiating a caspase cascade that eventually leads to cell death [87]. Caspases are intracellular proteases which are activated in a sequential manner and result in the formation of apoptotic bodies. Caspases 3 and 7 are effector caspases which execute apoptosis amongst other caspases [88]. The outcome of caspases includes the formation of apoptotic bodies, expression of ligands for phagocytic cell receptors and uptake of the apoptotic bodies by phagocytic cells [89]. In our study, we found that ZnONPs effectively enhanced the ROS level in HepG2 cells, which caused a loss of MMP. Furthermore, ZnONPs enhanced the activity of caspase-3 and caspase-7, which eventually led to cancer cell death as evident from the lower cell viability in HepG2 cells treated with ZnONPs as compared to NTCs. Exceptionally, the green-synthesized ZnONPs under UV-C light showed more promising anti-cancerous activity owing to the role UV-C light played in tuning the size and morphology of ZnONPs.

### 3.6. Biocompatibility Studies of Green-Synthesized ZnONPs

#### 3.6.1. Brine Shrimp Lethality Assay

**Figure 9:**
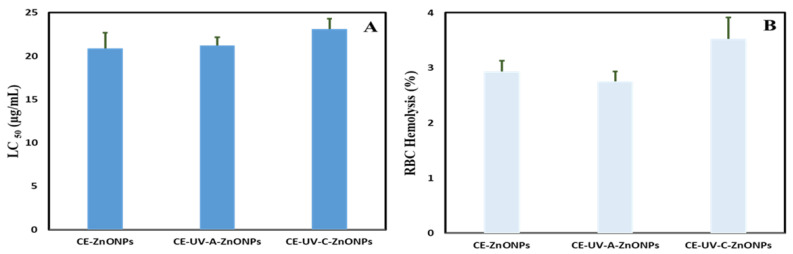
Biocompatibility studies of green-synthesized ZnONPs (**A**) Lethality of ZnONPs against brine shrimp (**B**) Biocompatibility of ZnONPs with human red blood cells (hRBCs).

Brine shrimp help the study of the toxicological effects of NPs on living organisms, being a low-cost and simple method. Moreover, *Artemia* are available throughout the year, are easy to culture, have a short life cycle and high offspring production rate and do not need feeding during the assay [90]. A well-known chemotherapeutic agent, doxorubicin, was used as a control, presenting an LC_50_ value of 5.92 µg/mL. Compounds showing LC_50_ between 10.0–30.0 µg/mL are interpreted as moderately toxic compounds [33]. In our study, the green-synthesized ZnONPs were found to be moderately toxic: CE-ZnONPs LC_50_ value was 20.90 ± 1.78 µg/mL, CE-UV-A-ZnONPs LC_50_ value was 21.20 ± 0.96 µg/mL and CE-UV-C-ZnONPs LC_50_ value was 23.13 ± 1.19 µg/mL (Figure 9A). Abbasi et al. [70] also reported the moderately toxic nature of green-synthesized ZnONPs (51.7 nm) against brine shrimp. The dose-dependent cytotoxicity of ZnONPs synthesized from *Hyphaene thebaica* towards brine shrimp was described by Mohamed et al. [91]. Dobretsov et al. [92] showed that the toxicity of ZnONPs towards *Artemia salina* depends on the morphology and concentration of ZnONPs, where ZnO nano-rods (width 80 nm) were least toxic. Our results showed the moderately toxic nature of ZnONPs at 300 µL concentration, which could be further reduced at lower concentrations.

#### 3.6.2. Biocompatibility with Human Red Blood Cells (hRBCs)

The biocompatible nature of ZnONPs was assessed with hRBCs in order to find out the percentage of hemolysis. All ZnONPs were found to be slightly hemolytic as their hemolysis value was between 2–5% based on the standards of the American Society for Testing and Materials Designation [93]. We found the slightly hemolytic nature of ZnONPs as CE-ZnONPs caused 2.94 ± 0.19% hemolysis, CE-UV-A-ZnONPs caused 2.75 ± 0.18% while CE-UV-C-ZnONPs caused greater hemolysis, i.e., 3.52 ± 0.39% (Figure 9B). The higher percentage of hemolysis caused by CE-UV-C-ZnONPs could be attributed to their small size as compared to other ZnONPs. We can conclude that ZnONPs are moderately biocompatible as reported previously [70]. Kumar et al. [93] also reported 2.7–6.4% hemolysis of RBCs when subjected to 100 and 500 µg/mL of green-synthesized ZnONPs from *S. gluaca*. Here we can conclude that our ZnONPs synthesized from plant extract are biocompatible for use in humans as alternate drugs since 5% hemolysis is permissible for biomaterials [94].

## 4. Conclusions

We have reported the synthesis of ZnONPs by a green and environmentally friendly method using *Casuarina equisetifolia* leaf extract under the influence of UV-A and UV-C light for the first time. The phytochemical profile of *C. equisetifolia* showed that the plant is enriched with phenolic and flavonoid contents, hence showing increased antioxidant activity. FTIR spectra showed the presence of polyphenolic, carboxylic acid and aromatic compounds on the surface of ZnONPs, showing their possible use in the reduction of zinc oxide, due to their antioxidant properties, to form ZnONPs and their subsequent stabilization. SEM-EDX and XRD characterization established that UV-C light has a moderate role in controlling the morphology of ZnONPs and thus the physico-chemical properties that impact their subsequent applications. In this study, interestingly, CE-UV-C-ZnONPs showed excellent anti-bacterial and anti-cancerous activities. All ZnONPs were moderately hemolytic towards hRBCs and moderately toxic to brine shrimp, hence showing their reasonably bio-safe nature. Here, the role of UV-irradiation in the green synthesis of ZnONPs and ultimately the enhancement of the in vitro biological activities of ZnONPs has been described for the first time. In future, the effect of UV light on the green synthesis of nanoparticles can be further optimized for the large-scale synthesis of high-quality biocompatible ZnONPs for biological applications.

## Figures and Tables

**Table 1:** Zone of inhibition of green-synthesized ZnONPs against different bacterial strains.

Bacterial Strain	Sample Type	Mean of Zone of Inhibition (mm) (15 μL/well)		
		CE-ZnONPs	CE-UV-A-ZnONPs	CE-UV-C-ZnONPs
*Bacillus subtilis*	Negative Control	0	0	0
	Positive Control (ZnAc)	11	11	10
	ZnONPs	11	10	12
	Standard Amp disc	8	8	8
*Pseudomonas fluorescens*	Negative Control	1	1	1
	Positive Control (ZnAc)	10	10	10.5
	ZnONPs	9	8.5	9.5
	Standard Amp disc	7	7.5	8.5
*Pseudomonas aeruginosa*	Negative Control	1	1	1
	Positive Control (ZnAc)	9	9	9
	ZnONPs	10.5	12	15
	Standard Amp disc	7	8	7

## Data Availability

All data are included in present study.
